# Molecular investigation of tick-borne pathogens in *Ixodes ricinus* feeding on lizards in the Central Pyrenees, Spain

**DOI:** 10.1007/s00436-026-08742-x

**Published:** 2026-08-21

**Authors:** Sofia M. Soares, Marta Sánchez-Sánchez, Ruth Rodríguez-Pastor, Clara Muñoz-Hernández, Alberto Moraga-Fernández, Laura Colorado, Henar Alonso, María Paz Peris, Patrick S. Fitze, Isabel G. Fernández de Mera, Javier Millán

**Affiliations:** 1 Instituto Agroalimentario de Aragón-IA2 (Universidad de Zaragoza-CITA), Zaragoza, Spain; 2 SaBio, Instituto de Investigación en Recursos Cinegéticos, IREC (CSIC-UCLM- JCCM), Ciudad Real, 13005 Spain; 3 Museo Nacional de Ciencias Naturales (MNCN-CSIC), Madrid, Spain; 4 Departamento de Microbiología, Pediatría, Radiología y Salud Pública, Facultad de Medicina, Universidad de Zaragoza, Zaragoza, 50009 España; 5 Departamento de Patología Animal, Facultad de Veterinaria, Universidad de Zaragoza, Zaragoza, Spain; 6 Instituto Pirenaico de Ecología (IPE-CSIC), Jaca, Huesca Spain; 7 Fundación ARAID, Avda. Ranillas 1, Zaragoza, 50018 Spain; 8 One Health Institute, Facultad de Ciencias de la Vida, Universidad Andrés Bello, Santiago, Chile

**Keywords:** Alpine, Lyme disease, Reptile, *Rickettsia*

## Abstract

*Ixodes ricinus* is the most widespread tick species in Europe, parasitizing a broad range of vertebrate hosts, including reptiles. However, the role of lizards in the ecology of tick-borne pathogens remains poorly understood. This study investigated tick infestation levels and the occurrence of tick-borne pathogens in *I. ricinus* feeding on European common lizards *(Zootoca vivipara louislantzi)* in the central Pyrenees, Spain. A total of 137 lizards were captured in summer and spring of 2024 at two sites (~ 1700 m.a.s.l.). Tick infestation prevalence was 98.5% and a mean abundance of 5.8 ± 7.0 ticks. Infestation was significantly higher in males, adults, and during spring. Molecular screening of 525 ticks (188 pools) using real-time and conventional PCR protocols detected *Rickettsia* spp. DNA in 15.4% pools, with an overall minimum infection rate (MIR) of 5.52%, with stage-specific MIRs of 4.79% in larvae and 7.81% in nymphs. Sequencing of 14 amplicons identified *Rickettsia helvetica* (*n* = 11) and *Rickettsia monacensis* (*n* = 3). *Babesia* DNA was detected at low prevalence (MIR: 0.38%) and sequences clustered within the *Babesia divergens-Babesia capreoli* complex. No *Borrelia burgdorferi* sensu lato and *Anaplasma phagocytophilum* DNA was detected. Our findings show that lizards can sustain high levels of *I. ricinus* infestation, constituting a reliable bloodmeal source for ticks infected with *Rickettsia* and *Babesia* in the mountain ecosystems of the Pyrenees. Although the lizard’s role in pathogen transmission remains to be clarified, its consistent exposure to immature ticks highlights its potential value as a sentinel species for monitoring tick-borne pathogen circulation in high-altitude environments.

**Supplementary Information:**

The online version contains supplementary material available at https://doi.org/10.1007/s00436-026-08742-x.

## Highlights

High infestation levels of *Ixodes ricinus* were recorded on the European common lizard from high-altitude sites in the Pyrenees.

Infestation patterns varied significantly by host sex, age and season.

*Rickettsia helvetica*, *Rickettsia monacensis* and *Babesia divergens-Babesia capreoli c*omplex were detected in ticks feeding on lizards.

No DNA of other major tick-borne pathogens screened (including *Borrelia burgdorferi* sensu lato and *Anaplasma phagocytophilum*) was detected.

**Supplementary Information:**

The online version contains supplementary material available at https://doi.org/10.1007/s00436-026-08742-x.

## Introduction

*Ixodes ricinus* is the most widespread tick species in Europe, displaying a broad host range, with immature stages commonly parasitizing small mammals, birds, and reptiles (Kahl and Gray 2023). Although the ecology of *I. ricinus* has been extensively studied, the role of reptiles has historically been underestimated compared with other hosts. However, studies have reported high levels of tick infestation in lizards, suggesting that reptiles may play a more significant role in the tick’s life cycle than previously recognized (Amore et al. 2007; Tomassone et al. 2017). Beyond serving as bloodmeal hosts, lizards may also serve as reservoirs for tick-borne pathogens (Dsouli et al. 2006; De Sousa et al. 2012; Norte et al. 2015). Notably, *I. ricinus* feeding on lizards has been shown to harbor multiple pathogens, including *Borrelia* spp., *Rickettsia* spp. and *Anaplasma* spp. emphasizing their potential epidemiological importance (Ekner et al. 2011; Václav et al. 2011; Tomassone et al. 2017).

The *Borrelia burgdorferi* sensu lato (s.l.) complex represents the most widespread group of tick-borne bacteria in Europe and includes several species associated with reptiles. In ticks collected from lizards, different members of this complex have been identified, but only *Borrelia lusitaniae* has been detected in lizard tissue samples (Majláthová et al. 2006, 2008; Földvári et al. 2009; Tijsse-Klasen et al. 2010). Consequently, it is considered to be maintained within natural cycles involving lizards as reservoirs and *I. ricinus* as the main vector (Amore et al. 2007; Norte et al. 2015). This potentially pathogenic species (Collares-Pereira et al. 2004) has been detected in questing ticks in the north-western regions of Spain (Díaz et al. 2017; Remesar et al. 2019a, b), although it has not yet been reported in Iberian lizards.

In Europe, *Rickettsia monacensis* and *R. helvetica*, both members of the spotted fever group (SFG), have been detected in ticks feeding on lizards as well as in lizard tissues, suggesting that reptiles may act as reservoir hosts (Tijsse-Klasen et al. 2010; De Sousa et al. 2012; Tomassone et al. 2017). Nevertheless, studies investigating the role of lizards in the ecology of *Rickettsia* remain limited and are especially scarce in the Iberian Peninsula (De Sousa et al. 2012; Kubelová et al. 2015; Tomassone et al. 2017; Mendoza-Roldan et al. 2021).

Other tick-borne bacteria have occasionally been associated with European lizards, including some Anaplasmataceae (Ekner et al. 2011; Köhler et al. 2023; Václav et al. 2011). The presence of *Ehrlichia* spp. and *Coxiella burnetii* was investigated in ticks attached to reptiles in Italy, but only a single tick tested positive for *Ehrlichia* sp.(Mendoza-Roldan et al. 2021).

Moreover, tick-borne protozoa have received even less attention in studies of lizard-tick associations. In particular, *Babesia* spp., which comprise several species transmitted by *I. ricinus* (Gray et al. 2019), are usually not investigated in ticks feeding on lizards. Only a few studies have addressed this topic, including the pathogen screening of ticks collected from sand lizards (*Lacerta agilis*) in the Netherlands (Tijsse-Klasen et al. 2010; Köhler et al. 2023), where *Babesia* was either not detected or detected at very low prevalence.

The European common lizard or viviparous lizard (*Zootoca vivipara*) is the most widely distributed terrestrial reptile worldwide, with Iberian populations located in the southwest of its range, where it frequently inhabits montane ecosystems (Horreo et al. 2021). This species exhibits a relatively small home range, approximately 15 m in radius, with variation by age and sex (Lecomte et al. 1994; Massot and Clobert 2000). Such limited spatial movement makes it a useful model for studying local interactions between hosts, ticks, and the pathogens they harbor. Given the overlap between the habitats occupied by *I. ricinus* and the common lizard, this species may play a significant role in the maintenance of ticks and the pathogens they carry.

The Pyrenees constitute a mountainous region, where *I. ricinus* is abundant (Alonso-Carné et al. 2016; Estrada-Peña 2001). However, data from the Spanish Pyrenees remain scarce, particularly regarding reptile-associated tick-pathogens. In this study, we analyzed *I. ricinus* infestation levels in the European common lizard *(Zootoca vivipara louislantzi)* and used feeding ticks to investigate the presence of vector-borne pathogens in the central Pyrenees, Spain.

## Materials and methods

### Field methods

The study was carried out in the central Pyrenees at two sites in the Huesca province (Aragón Autonomous Region): site 1 in Somport (42°47’N, 0°31’W) and site 2 in Candanchú (42°46’N, 0°32’W), located at approximately 1650 m and 1680 m above sea level (a.s.l.), respectively. The study sites were purposefully selected due to the presence of monitored populations of the common lizard known to be infested with ticks (Peñalver-Alcázar et al. 2016).

The sites are characterized by meadows and grasslands with a predominance of herbs and small shrubs (Peñalver-Alcázar et al. 2016), being popular year-round tourist destinations for hiking, climbing and skiing. Lizards were captured by hand (Peñalver‐Alcázar et al. 2016), during two sampling sessions in 2024: one conducted in late spring (June) at site 1 and in summer (August) in both sites. A total of 137 common lizards were examined, including 33 captured in June and 104 in August, with 112 at site 1 and 25 at site 2. Each captured lizard was characterized by sex and age, as well as classified as either adult (> 2 years) or juvenile (< 2 years) (Le Galliard et al. 2005). The distribution of individuals by site, season, age class and sex is presented in Table 1.

**Table 1:** Number of common lizards (*Zootoca vivipara louislantzi*) captured by site, season, age and sex groups in the Central Pyrenees, Spain. Two individuals could not be assigned to sex and are included in the total counts

Site	Season	Age class	Female	Male
Site 1 (Somport)	Spring	Adult	13	8
		Juvenile	7	3
	Summer	Adult	31	13
		Juvenile	19	16
Site 2 (Candanchú)	Summer	Adult	15	0
		Juvenile	10	0

Attached ticks were carefully removed using tweezers and placed into plastic vials containing 95% ethanol and stored at room temperature. After careful examination, each lizard was released at its original capture site. Tick species and life-stage were determined using the morphological keys on Estrada-Peña et al. (2017).

### Molecular methods

A total of 801 ticks (662 larvae and 139 nymphs) were collected. For pathogen screening, ticks were grouped into separate pools of larvae and nymphs (maximum of five ticks per pool) from the same host. A maximum of one pool per lizard and life stage was included. This resulted in 525 ticks (397 larvae and 128 nymphs) distributed in 188 pools (156 pools of larvae and 32 pools of nymphs).

Tick pools were first homogenized in a 0.2-ml sterile microtube containing ten 0.1-mm zirconia microbeads and one 3.2-mm sterile stainless steel microbead (BioSpec, Bartlesville, OK, USA) in a Mini Bead Beater-16 (Model 607-EUR, BioSpec, Bartlesville, OK, USA) for two cycles of one minute and thirty seconds at 3450 rpm. Genomic DNA was then extracted using the QIAGEN DNeasy Blood & Tissue Kit (Qiagen, Valencia, CA, USA), following the manufacturer’s instructions. The final elution volume was 100 µl.

All samples were initially screened for tick-borne pathogens using the VIASURE Tick-Borne Diseases panel from CerTest Biotec (Spain), a probe-based multiplex real-time PCR provided in two 8-well strip assays and including an internal control (IC) to monitor amplification performance and potential inhibition. The panel allows the detection of *Borrelia burgdorferi* s.l./*Borrelia miyamotoi* and/or *Borrelia hermsii* (23 S rRNA), *Anaplasma phagocytophilum* (*msp2*), *Coxiella burnetii* (IS1111), *Rickettsia* spp. (23 S rRNA), *Babesia microti* (CCT-eta), *Babesia divergens* (*hsp70*), and *Ehrlichia chaffeensis*/*Ehrlichia muris* (*groEL*) using specific primers and fluorescent-labelled hydrolysis probes.

Each reaction was run in a final volume of 20 µL per well, prepared by adding 15 µL of Rehydration Buffer to each lyophilized well and 5 µL of extracted DNA (samples or controls). A positive control and a negative (no-template) control were included in each run. The thermal profile consisted of 95 °C for 2 min, followed by 45 cycles of 95 °C for 10 s and 60 °C for 50 s, with fluorescence acquisition during the 60 °C step. Amplification was performed on the CFX Opus 96 Real-Time PCR System (Bio-Rad, USA), and data were analyzed using Bio-Rad CFX Maestro software (version 2.3). Result interpretation followed the manufacturer’s criteria: a sample was considered positive when it showed a typical sigmoidal amplification curve with Ct < 40 in the corresponding target channel, with a valid IC signal when applicable.

Subsequently, all DNA pools were screened for *Rickettsia* spp. by conventional PCR (cPCR), targeting a 416-bp fragment of the 16 S ribosomal RNA (*16 S rRNA*) gene, independently of the real-time PCR results. Samples that scored positive were further confirmed and characterized by *Rickettsia*-specific conventional PCR assays amplifying recombinase A (*recA*) and adenosine triphosphate synthase alpha subunit (*atpA*) gene fragments (Table 2). In contrast, only DNA pools that tested positive for *B. microti*/*B. divergens* real-time PCR were subjected to cPCR for molecular characterization by amplifying a partial fragment of the 18 S ribosomal (*18 S rRNA*) gene (Table 2). For all cPCR assays, the reaction mixture consisted of 2X PCR Master Mix (Promega Corporation, USA), 1 µL of each primer at 10 µM, 1 µL of DNA, and nuclease-free water, totaling 25 µL. Primers and thermocycling conditions are detailed in Table 2, and all assays were carried out in a Bio-Rad C1000 Touch Thermal Cycler (Bio-Rad, USA). Amplicons were visualized in 1.5% agarose gels stained with GelRed (Biotium, USA). A pool was considered positive to *Rickettsia* spp. if tested positive to *16 S rRNA*, *atpA* and *recA* assays. Bands at the expected size for *recA* and *atpA* (*Rickettsia* spp.) and *18 S rRNA*f (*Babesia* spp.) gene fragments were sent for purification and Sanger sequencing (Macrogen, Spain). Chromatograms were visualized using Chromas 2.6.6 software (Technelysium Pty Ltd, Australia) to edit nucleotide sequences. The level of similarity between the nucleotide sequences obtained in this study and those deposited in the NCBI GenBank database was evaluated using the Nucleotide BLAST tool (https://blast.ncbi.nlm.nih.gov/Blast.cgi).

**Table 2:** Primers and conditions used for the amplification and characterization of *Rickettsia* spp. and *Babesia* spp. in *Ixodes ricinus* feeding on common lizards (*Zootoca vivipara louislantzi*) in the Central Pyrenees, Spain

Gene target^1^	Primers		Size (bp)	Annealing conditions	Reference
	Name	Sequence (5’ – 3’)			
*Rickettsia spp.*					
16 S ribosomal RNA (*16 S rRNA*)	fD1	AGAGTTTGATCCTGGCTCAG	416	30 s at 54 °C	Weisburg et al. (1989)
	Rc16	AACGTCATTATCTTCCTTGC			
Recombinase A gene (*recA*)	F	TGCTTTTATTGATGCCGAGC	428	30 s at 52 °C	Fernández de Mera et al. (2009)
	R	CTTTAATGGAGCCGATTCTTC			
Adenosine triphosphate synthase alpha subunit gene (*atpA*)	F	ACATATCGAGATGAAGGCTCC	731	30 s at 48 °C	Fernández de Mera et al. (2009)
	R	CCGAAATACCGACATTAACG			
*Babesia spp.*					
18 S ribosomal RNA (18 S rRNA)	PIRO F	CCAGCAGCCGCGGTAATTC	360	45 s at 64 °C	Tabar et al. (2008)
	PIRO R	CTTTCGCAGTAGTTYGTCTTTAACAAATCT			

A Maximum Likelihood phylogenetic tree was generated using Molecular Evolutionary Genetics Analysis 10 (MEGA X) software to confirm the species identification of *Rickettsia* spp. (Kumar et al. 2018). Concatenated partial fragments of *atpA* and *recA* genes were used to construct the phylogenetic tree, which allowed for improving phylogenetic resolution and robustness (Vitorino et al. 2007). Alignments of our nucleotide sequences, together with reference sequences retrieved from GenBank, were performed using the ClustalW tool implemented in Ugene software. The evolutionary history was inferred using the Tamura 3-parameter substitution model, which had the lowest Bayesian Information Criterion (BIC) values. A discrete Gamma distribution was used to model evolutionary rate differences among sites. Statistical support for tree topology was assessed through bootstrap analysis with 1000 replicates.

### Statistical analysis

A descriptive analysis was conducted to calculate the prevalence of infestation with 95% Clopper-Pearson confidence intervals, the mean tick abundance and the mean tick intensity, each reported with the corresponding standard deviation. All metrics were calculated separately for larvae, nymphs, and overall infestation. Parasitological terms (e.g., prevalence, mean abundance and mean intensity) follow the standardized definitions proposed by Bush et al. (1997).

Tick abundance was analysed using generalized linear models (GLMs) with a negative binomial distribution, using separate models for total, larval, and nymphal tick infestation. Predictor variables included sex (female, male), age class (juvenile, adult), and a combined site–season factor to control for potential confounding effects between these variables, since site 2 was sampled only in summer. Three individuals for which sex could not be determined were excluded from the analysis, resulting in a total sample size of 134 lizards. To explicitly test for seasonal differences, an additional model restricted to site 1 (the only site sampled in both spring and summer) was fitted with sex, age, and season as predictors. Model coefficients were expressed as incidence rate ratios (*IRR*) with 95% confidence intervals, and statistical significance was set at *p* < 0.05 for all tests.

Agreement between the two screening PCR protocols for *Rickettsia* spp. was evaluated using Cohen’s kappa coefficient to assess concordance beyond chance. The coefficient was calculated to compare the presence or absence of *Rickettsia* spp. detected by both methods. Kappa values were interpreted according to the guidelines for agreement proposed by Mary L. McHugh (2012).

All statistical analyses were performed in R (version 4.4.0; R Core Team, 2024).

For assessing infection levels in larvae and nymphs, the Minimal Infection Rate (MIR) was calculated under the assumption that each positive pool contained only one infected tick (Estrada-Peña et al. 2021). In the case of *Rickettsia* spp., the MIR calculated was based on the cPCR results.

## Results

### Parasite ecology

Overall, 135 out of 137 lizards were infested with ticks (prevalence = 98.5%; 95% CI: 94.8–99.8%). All ticks were larvae or nymphs of *I. ricinus*, with 54 lizards infested with both stages simultaneously. In total, 801 ticks were collected and identified, of which 662 (83%) were larvae and 139 (17%) were nymphs. Mean tick abundance was 5.8 ± 7.0, and the mean intensity was 5.9 ± 7.0, with a maximum of 41 ticks recorded on a single host (Table 3).

**Table 3:** Description of the infestation by *Ixodes ricinus* in 137 common lizards (*Zootoca vivipara louislantzi*) captured in the central Pyrenees

Tick stage	Larvae	Nymphs	Overall
% Prevalence (95% CI)	91.9 (86.1–95.9)	45.9 (37.4–54.7)	98.5 (94.8–99.8)
Range	*0–37*	*0–12*	*0–41*
Mean abundance ± SD	4.8 ± 6.1	1.0 ± 1.7	5.8 ± 7.0
Mean intensity ± SD	5.3 ± 6.2	2.2 ± 1.9	5.9 ± 7.0

The outcomes of the negative binomial GLMs indicated significant effects of sex and season on total tick abundance (Table 4). Males had higher tick infestations than females (*IRR* = 1.43, 95% CI = 1.07–1.91, *p* = 0.017), while adults showed a higher estimated tick abundance than juveniles, although this difference did not reach statistical significance (*IRR* = 1.31, 95% CI = 0.98–1.74, *p* = 0.052). Moreover, tick infestation was significantly lower in summer than in spring (*IRR* = 0.37, 95% CI = 0.27–0.51, *p* < 0.001).

**Table 4:** Results of the negative binomial generalized linear models (GLMs) assessing the effects of sex, age, and site–season on total, larval and nymphal tick infestation. Model estimates (β) with their standard errors (SE) are presented on the log scale. Incidence Rate Ratios (*IRR*s) are the exponentiated estimates with 95% confidence intervals (CI)

Model	Predictor	β	SE	z value	*p*-value	*IRR*	95% CI
Total tick abundance	(Intercept)	2.169	0.171	12.7	< 0.001	8.75	6.03–12.81
	Sex (Male vs. Female)	0.354	0.148	2.39	0.017	1.43	1.07–1.91
	Age (Adult vs. Juvenile)	0.268	0.138	1.95	0.052	1.31	0.98–1.74
	Site-season (Site 1 Summer vs. Site 1 Spring)	-1.035	0.153	-6.76	< 0.001	0.36	0.26–0.49
	Site-season (Site 2 Summer vs. Site 1 Spring)	-1.165	0.215	-5.42	< 0.001	0.31	0.20–0.48
Larval abundance	(Intercept)	1.99	0.198	10.05	< 0.001	7.31	4.74–11.43
	Sex (Male vs. Female)	0.373	0.171	2.18	0.029	1.45	1.04–2.04
	Age (Adult vs. Juvenile)	0.193	0.158	1.22	0.222	1.21	0.87–1.68
	Site-season (Site 1 Summer vs. Site 1 Spring)	-0.983	0.178	-5.51	< 0.001	0.37	0.26–0.54
	Site-season (Site 2 Summer vs. Site 1 Spring)	-1.062	0.247	-4.31	< 0.001	0.35	0.21–0.57
Nymphal abundance	(Intercept)	0.186	0.267	0.7	0.486	1.2	0.69–2.05
	Sex (Male vs. Female)	0.312	0.221	1.41	0.159	1.37	0.89–2.10
	Age (Adult vs. Juvenile)	0.829	0.243	3.41	< 0.001	2.29	1.43–3.79
	Site-season (Site 1 Summer vs. Site 1 Spring)	-1.309	0.218	-6.01	< 0.001	0.27	0.18–0.41
	Site-season (Site 2 Summer vs. Site 1 Spring)	-1.774	0.393	-4.52	< 0.001	0.17	0.07–0.35

Note: Reference categories used in the models were: female (sex), juvenile (age), spring (season), Site 1–Spring (site–season)

When modelled separately, male lizards carried more larvae than females (*IRR* = 1.45, 95% CI = 1.04–2.04, *p* = 0.029), and larval tick infestations were lower in summer (*IRR* = 0.37, 95% CI = 0.26–0.54, *p* < 0.001). Age was not significantly associated with larval infestation (*p* > 0.05). Adult lizards hosted more nymphs than juveniles (*IRR* = 2.29, 95% CI = 1.43–3.79, *p* < 0.001) and nymphal tick infestation was lower in summer than in spring (Site 1 Summer: *IRR* = 0.27; *p* < 0.001). No significant association was found between host sex and nymph tick infestation (*p* > 0.05).

### Pathogen detection and identification

The qPCR detected *Rickettsia* spp. DNA in 30/188 pools (16.0%), from 28 individual lizards, comprising 17 pools of larvae and 13 pools of nymphs. According to the cPCR, *Rickettsia* spp. DNA was detected in 29/188 pools (15.4%) belonging to 26 lizards, including 19 pools of larvae and 10 pools of nymphs. Overall MIR, based on cPCR-positive pools, was 5.52% (29/525, 95% CI: 2.6–9.5), whereas the MIRs for larvae and nymphs were 4.79% (19/397, 95% CI: 1.8–9.1) and 7.81% (10/128, 95% CI: 0.8–21.0), respectively. Among the 188 samples analyzed with both PCR protocols, 22 were positive in both assays, eight were positive only by qPCR and seven were positive only by cPCR, resulting in a total of 37 pools positive by at least one method. The overall agreement between methods was 92%, yielding a Cohen’s kappa value of 0.70 (95% CI: 0.56–0.84), indicating a moderate level of agreement between the two PCR methods.

Additionally, the qPCR protocol detected DNA of *B. microti/B. divergens* in 2/188 pools (1.1%) from two lizards, including one larval pool and one nymphal pool. The overall MIR for *Babesia* sp. was 0.38% (2/525; 95% CI: 0.10–1.38), while larval and nymphal MIRs were 0.25% (1/397; 95% CI: 0.04–1.41) and 0.78% (1/128; 95% CI: 0.14–4.29), respectively.

**Fig. 1:**
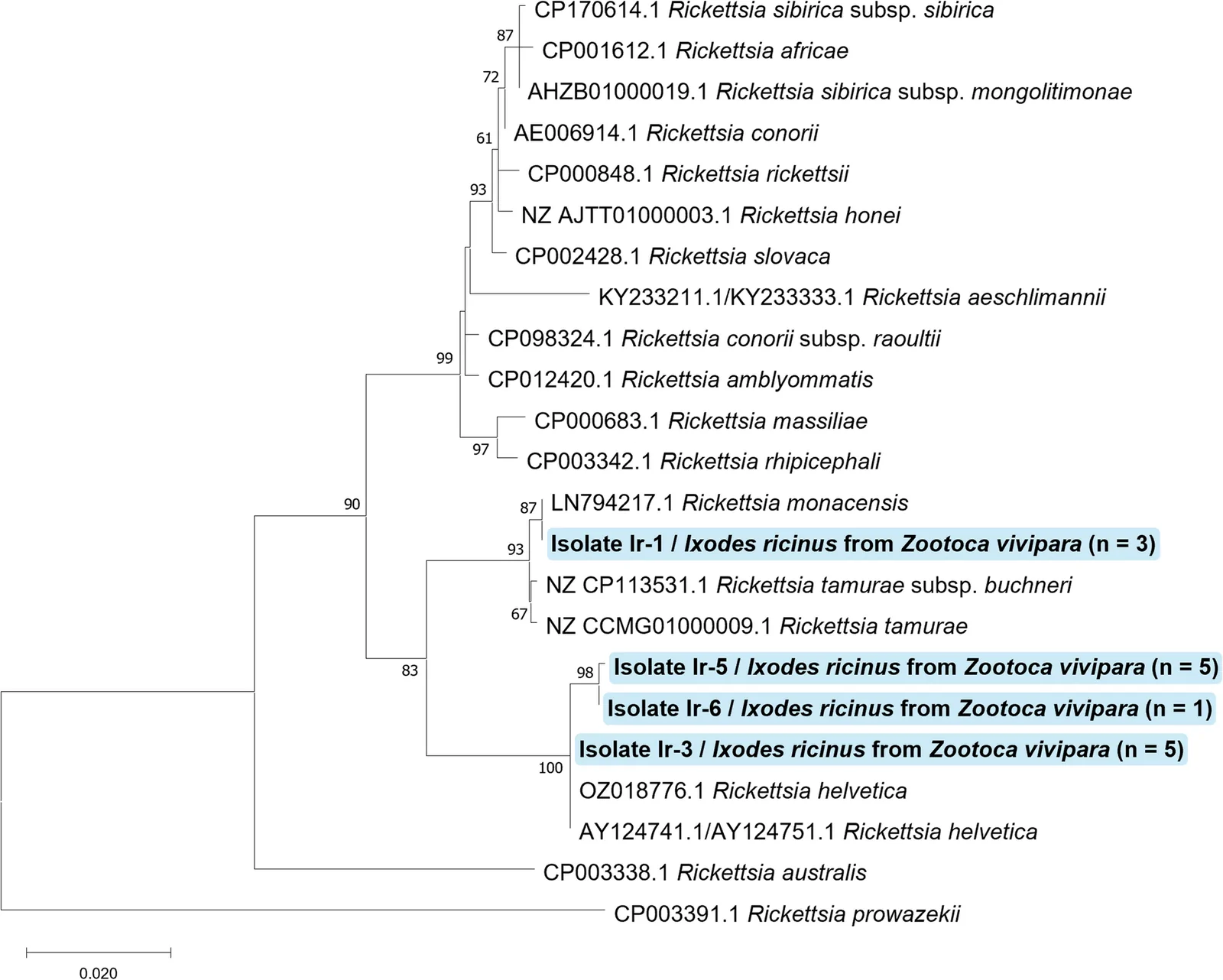
Maximum Likelihood phylogenetic tree of spotted Fever Group *Rickettsia* nucleotide sequences identified in this study (highlighted in bold and shaded in blue), together with reference sequences from GenBank. The tree was constructed using concatenated partial fragments of *atpA* and *recA* genes (1044 base pairs). *Rickettsia prowazekii* was used as an outgroup. Numbers at nodes indicate bootstrap support (values below 50% are not shown)

The sequencing of *recA* and *atpA* partial fragments from 14 randomly selected cPCR-positive pools confirmed the presence of *R. helvetica* and *R. monacensis*. *Rickettsia helvetica* DNA was detected in 11 pools (five pools of larvae and six pools of nymphs from 11 different lizards), and *R. monacensis* DNA was confirmed in three pools (two pools of larvae and one pool of nymphs from three lizards). The *recA* sequences obtained were 100% identical to those deposited in the GenBank database (LN794217.1, CP113531.1, OZ018776.1), while the identity for *atpA* sequences ranged from 99.3% to 100% (OZ018776.1). The concatenated *atpA* and *recA* fragments obtained from 14 pools and used for phylogenetic analysis yielded four distinct concatenated sequences. One of them were grouped with *R. monacensis*, while the remaining three were clustered with *R. helvetica* (Fig. 1). Unique partial sequences identified in this study were deposited in GenBank under the accession numbers PX596871 (Ir5_atpA) and PX596872 (Ir6_atpA).

On the other hand, partial *18 S rRNA* gene fragments were sequenced from two positive pools and the presence of *Babesia* spp. was confirmed. The sequences obtained showed 99.64% similarity between them and were 100% identical with those deposited in the GenBank database, corresponding to the *Babesia divergens* complex (*Babesia capreoli* -MG344755.1-; *Babesia* sp. FR1 -MZ825347.1-; *B. divergens* -MK256977.1-). Species-level discrimination within the *B. divergens* complex was not possible.

No DNA of other tick-borne pathogens targeted by the qPCR panel was detected in any of the analyzed pools, including *Borrelia* spp., *A. phagocytophilum*,* C. burnetii*,* and E. chaffeensis/E. muris.*

## Discussion

This study confirms that the common lizard serves as a host for the immature stages of *I. ricinus* and may locally sustain high levels of tick infestation in the central Pyrenees. We also demonstrated the presence of tick-borne *Rickettsia* spp. and *Babesia* sp. in ticks feeding on this lizard species. The detection of pathogen DNA in feeding ticks does not represent or constitute proof of vectorial competence, nor does it indicate that lizards are hosts of the detected pathogens (Estrada-Peña et al. 2021). However, our findings support that reptiles, particularly lacertid lizards, can be used for the detection of local circulation of tick-borne pathogens.

The prevalence of *I. ricinus* infestation observed in our study was remarkably high compared to previous reports for common lizards (Wu et al. 2019; Gwiazdowicz et al. 2020). Given that the studied lizard populations were selected due to their known high tick prevalence (Fitze, personal observations), they do not represent average infestation levels across the Pyrenees. Adult common lizards exhibited significantly higher nymphal infestation than juveniles. This may be due to their larger body size and wider home ranges, which increase both surface area for tick attachment and the likelihood of tick exposure (Bauwens et al. 1983; Perry and Garland 2002). Male individuals also carried significantly more ticks than females, which is consistent with previous reports (Dudek et al. 2016; Wu et al. 2019; Gwiazdowicz et al. 2020; Hernández Rojas et al. 2025). Such male-biased infestations are commonly explained by the greater activity and broader home ranges of males, particularly during the mating season (Bauwens et al. 1983; Perry and Garland 2002). In addition, hormonal factors may contribute to increased male susceptibility to ectoparasites, since elevated testosterone levels can suppress immune responses (Salvador et al. 1996). Notably, tick infestation was significantly higher in spring than in summer, coinciding with the common lizard’s reproductive season (Bleu et al. 2013) and the peak activity period of immature *I. ricinus* (Alonso-Carné et al. 2016).

*Rickettsia* spp. was the most prevalent pathogen detected in both larvae and nymphs. The presence of infected larvae may result from transovarial transmission, a common feature among *Rickettsia* species. This mechanism allows the bacteria to persist within tick populations without the need for an infected vertebrate host (Azad and Beard 1998). The MIRs observed in this study were lower than those reported elsewhere. For example, ticks feeding on common lizards in Poland showed MIRs of 23.2% in larvae and 35.3% in nymphs (Dyczko et al. 2024). Similarly, in the Central System Mountains of the Iberian Peninsula, 47% of nymphs and 32% of larvae collected from the Iberian emerald lizard (*Lacerta schreiberi*) were *Rickettsia*-positive (Kubelová et al. 2015). Such differences may reflect regional and ecological differences, host species variation, or the pooling of ticks in our study, which can underestimate true infection prevalence.

Sequencing revealed the presence of *R. helvetica* and *R. monacensis*, consistent with previous studies indicating *I. ricinus* as the main vector of both species and the frequent detection of these *Rickettsia* in *I. ricinus* ticks in the Iberian Peninsula (Márquez 2008; Milhano et al. 2010; Moerbeck et al. 2022). *Rickettsia helvetica* has previously been detected in common wall lizard (*Podarcis muralis*) tissues in northern Italy and in the Madeiran wall lizard (*Teira dugesii*) from Portugal (De Sousa et al. 2012; Tomassone et al. 2017), demonstrating that lizards can harbor the bacterium. Human infections caused by *R. helvetica* have been reported in France, Sweden, and Switzerland (Oteo and Portillo 2012). In Spain, this species has been identified in *I. ricinus* collected from humans (Fernández-Soto et al. 2006; Ibarrondo-Mendiola et al. 2025), but no human clinical cases have been reported to date. Similarly, *R. monacensis* has been detected previously in lizard tissues in Portugal (De Sousa et al. 2012) and in ticks parasitizing lizards (Kubelová et al. 2015; Tomassone et al. 2017). *Rickettsia monacensis* is a recognized human pathogen and has been reported in different European countries, including Spain (De Sousa et al. 2022; Jado et al. 2007; Portillo et al. 2015). In a recent study, *R. monacensis* was identified as the most prevalent *Rickettsia* species in ticks collected from humans in the Basque Country (Ibarrondo-Mendiola et al. 2025), underscoring its public health importance.

*Babesia* sp. was detected at low MIR, in concordance with previous studies analyzing questing ticks from the French Pyrenees (0.4%) and other European studies (Akl et al. 2019; Hamšíková et al. 2016; Onyiche et al. 2021; Rosso et al. 2024). Nevertheless, *Babesia* prevalence may be underestimated. The qPCR assays used were highly species-specific and may not have detected other circulating *Babesia* lineages, and only cPCR was performed in the tick pools positive for qPCR. Transovarial transmission and transstadial persistence have been documented for *B. divergens* (Bonnet et al. 2007) and, given the close genetic similarity between *B. divergens* and *B. capreoli*, similar transmission patterns are considered likely for the latter (Gray et al. 2019). These transmission routes may therefore partly explain the detection of *Babesia* DNA.

Although sequencing did not allow discrimination between *B. divergens* and *B. capreoli*, the detection of genotypes within this complex is consistent with previous reports from the Pyrenees and other regions of Spain (Akl et al. 2019; Remesar et al. 2019a, b). These two species are associated with partially distinct ecological cycles: *B. capreoli* is primarily maintained in wild cervids (Andersson et al. 2016; Remesar et al. 2019a, b), whereas *B. divergens* is historically linked to cattle (Hildebrandt et al. 2021), but is also detected in wild ungulates (Fanelli 2021; Leverett et al. 2025). In the study area, both wild boar (*Sus scrofa*) and roe deer (*Capreolus capreolus*) are abundant, alongside other wild ungulates (Herrero et al. 2013; González et al. 2013). This makes the circulation of these piroplasmids expected. Given that *B. divergens* is a recognized zoonotic agent and the main cause of human babesiosis in Europe (Hildebrandt et al. 2013). Therefore, the detection of *B. divergens–capreoli*–like genotypes suggests that potentially pathogenic *Babesia* spp. may circulate in high-altitude recreational areas of the Pyrenees. Nevertheless, the role of reptiles in the ecology of *Babesia* remains poorly understood. Most research on piroplasmids has focused on mammals, while reptiles and other non-mammalian vertebrates remain largely understudied (Schnittger et al. 2012). Similar low infection rates have been reported in ticks feeding on sand lizards (Tijsse-Klasen et al. 2010).

Notably, no DNA of other major tick-borne pathogens targeted by the VIASURE Tick-Borne Diseases panel (CerTest Biotec) was found. This contrasts with previous studies conducted in the Pyrenees, where a broader diversity of pathogens has been reported in questing *I. ricinus*, including *B. burgdorferi* s. l. and *A. phagocytophilum* (Akl et al. 2019 b). However, data on the presence of tick-borne pathogens in the central Pyrenees remain limited. Therefore, the absence of these pathogens in ticks feeding on common lizards may therefore reflect local ecological conditions or limited circulation of these agents in the studied area. It may also be influenced by the use of a targeted molecular assay designed for specific pathogens, potentially overlooking other circulating tick-borne agents.

Interestingly, no *Borrelia* DNA was detected in feeding *I. ricinus*. While several lizard species have demonstrated potential reservoir competence for *B. lusitaniae*, including the Spanish wall lizard (*Podarcis hispanicus*) in Portugal (Norte et al. 2015) and the common wall lizard in Tuscany, Italy (Amore et al. 2007). However, the role of the common lizard remains unclear. In Poland, *B. lusitaniae* DNA was detected in nymphs feeding on common lizards (Dyczko et al. 2024), suggesting possible involvement in local transmission cycles. The absence of *Borrelia* in our study may therefore reflect either limited local circulation of the pathogen or a lack of capacity of this lizard species to maintain *Borrelia* in natural systems.

In summary, our findings identify the common lizard as a frequent host of immature *I. ricinus* in high-altitude Pyrenean ecosystems and show that ticks feeding on this species harbor tick-borne pathogens. Although the reservoir competence of the common lizard for these pathogens remains undetermined, its consistent exposure to infected ticks supports its value as a sentinel species for monitoring local tick-borne pathogen circulation in mountain environments. Further studies are needed to clarify whether this reptile actively contributes to pathogen maintenance or primarily sustains infected tick populations.

## Supplementary Information

Below is the link to the electronic supplementary material.

Supplementary Material 1 (DOCX 3.99 MB)

## Data Availability

The sequence data obtained from this study have been deposited in the GenBank^®^ database (https://www.ncbi.nlm.nih.gov/genbank), under the accession numbers: PX596871 and PX596872.
