# Supplementary material for: Molecular investigation of tick-borne pathogens in Ixodes ricinus feeding on lizards in the Central Pyrenees, Spain
Source: Parasitol Res. 2026 Aug 21;125(1):114. doi: 10.1007/s00436-026-08742-x (PMC13615137; doi:10.1007/s00436-026-08742-x)
Supplement: Supplementary file 1 [file 436_2026_8742_MOESM1_ESM.docx]

**Figure S1.** Agarose gel electrophoresis of PCR products of the *16S rRNA* gene fragment of *Rickettsia* spp. MWM: molecular weight marker; CP: positive control; CN: negative control.

**Figure S2.** Agarose gel electrophoresis of PCR products of the *recA* gene fragment of *Rickettsia* spp. MWM: molecular weight marker; CP: positive control; CN: negative control.

**Figure S3.** Agarose gel electrophoresis of PCR products of the *atpA* gene fragment of *Rickettsia* spp. MWM: molecular weight marker; CP: positive control; CN: negative control.

**Figure S4.** Agarose gel electrophoresis of PCR products of the *18S rRNA* gene fragment of piroplasmids. MWM: molecular weight marker; CP: positive control; CN: negative control.
